# Water in Protic Ionic Liquid Electrolytes: From Solvent Separated Ion Pairs to Water Clusters

**DOI:** 10.1002/cssc.202100660

**Published:** 2021-07-12

**Authors:** Sascha Gehrke, Promit Ray, Timo Stettner, Andrea Balducci, Barbara Kirchner

**Affiliations:** ^1^ Mulliken Center for Theoretical Chemistry University of Bonn Beringstr. 4+6 D-53115 Bonn Germany; ^2^ Department of Physics and Astronomy University College London London WC1E 6BT United Kingdom; ^3^ Institute for Technical Chemistry and Environmental Chemistry Friedrich-Schiller-University Jena Philosophenweg 7a D-07743 Jena Germany; ^4^ Center for Energy and Environmental Chemistry Jena (CEEC Jena) Friedrich-Schiller-University Jena Philosophenweg 7a D-07743 Jena Germany

**Keywords:** molecular dynamics, ionic liquids, water-in-salt, hydrogen bonds, electrolytes

## Abstract

The large electrochemical and cycling stability of “water‐in‐salt” systems have rendered promising prospective electrolytes for batteries. The impact of addition of water on the properties of ionic liquids has already been addressed in several publications. In this contribution, we focus on the changes in the state of water. Therefore, we investigated the protic ionic liquid *N*‐butyl‐pyrrolidinium bis(trifluoromethanesulfonyl)imide with varying water content at different temperatures with the aid of molecular dynamics simulations. It is revealed that at very low concentrations, the water is well dispersed and best characterized as shared solvent molecules. At higher concentrations, the water forms larger aggregates and is increasingly approaching a bulk‐like state. While the librational and rotational dynamics of the water molecules become faster with increasing concentration, the translational dynamics are found to become slower. Further, all dynamics are found to be faster if the temperature increases. The trends of these findings are well in line with the experimental measured conductivities.

## Graphical abstract

**From single molecules to bulk liquid**: The state of water in a protic ionic liquid at different concentration is investigated by the aid of molecular dynamics simulations. At a low concentration, the water shows the behavior of free molecules. With increasing concentration, the formation of clusters can be observed. Finally, at concentrations near the phase separation the behavior approaches that of the bulk liquid.

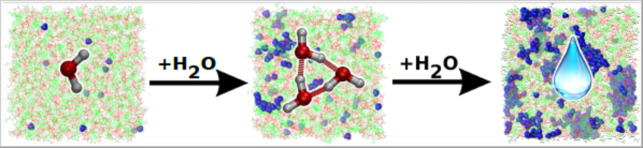

## Introduction

Ionic liquids (ILs) have attracted great attention as alternative solvents for a wide variety of technological applications well beyond just the solvation of molecules.[^1^, ^2^, ^3^, ^4^] Their inherent ionic conductivity – a virtue of their ionic nature – renders ILs as promising candidates for potential electrolytes in a variety of electrochemical devices.[^5^, ^6^, ^7^, ^8^, ^9^] However, some disadvantageous properties, their relatively high viscositiy[^7^, ^10^] in particular, strongly limit their use. It has been shown, that one way to tackle this problem is the addition of molecular liquids.[^11^, ^12^, ^13^, ^14^] Thereby, water was revealed to be the most promising candidate to optimize the ILs properties in terms of the application as electrolytes by the enhancement of the diffusivity of the ions.[^12^, ^15^, ^16^, ^17^, ^18^, ^19^] Unfortunately, water has a low electrochemical window of 1.23 V which restricts the applicability of water‐based electrolytes. However, it has been shown that the behavior of water differs significantly if the amount of ionic liquid clearly outnumbers the amount of water. The so‐called water‐in‐salt systems are characterized by a large electrochemical and cycling stability.[^20^, ^21^, ^22^] Moreover, extending studies based on aprotic ionic liquids[^7^, ^23^, ^24^, ^25^, ^26^] to more protic ionic liquids (PILs)[^27^, ^28^, ^29^] has shown to be an important preliminary step as PILs often exhibit enhanced conductivities on account of their water‐like hydrogen bonding networks.[^30^, ^31^, ^32^, ^33^]

The impact of added water on the structure and properties of ILs has already been addressed in several studies[^13^, ^34^, ^35^] highlighting differences between aprotic ILs and PILs. For the latter, the concept of contact ion pair, shared solvent ion pair, and separated ion pair[^36^, ^37^, ^38^, ^39^] could explain the change in the ions’ state in dependence of the concentration of water.^[40]^ Furthermore, with the aid of spectroscopic methods supported by computer studies, it has been shown that water molecules tend to be isolated at lower concentration but form a nanosegregated continuous water network at higher concentrations.[^12^, ^13^, ^35^, ^41^, ^42^, ^43^] However, details about the changes of the water's state – especially according to the formation of water‐in‐salt systems – are still largely a mystery.

In recent works, we already investigated the protic ionic liquid *N*‐butyl‐pyrrolidinium bis(trifluoromethanesulfonyl)imide ([pyrH4][NTf_2_]) as a promising potential electrolyte for lithium‐ion batteries[^44^, ^45^, ^46^] and characterized the impact of the addition of water on the properties of the ILs.^[47]^ This paper subsequently enhances the recent studies by giving closer attention to the changes in the water molecules’ state. Therefore, systems with water contents of 0.1 %, 1.0 %, 2.0 %, and 3.8 % by weight are investigated at different temperatures at and above room temperature. First, the simulation methodologies and experimental measurements are introduced, followed by a detailed analysis of the structural aspects of these solutions which we round up with a thorough discussion of the dynamic properties.

## Computational Details

The [NTf_2_]^−^ anion as well as the heterocyclic atoms of the cation were modeled using the specifically parametrized Canongia Lopes−Pádua force field.^[48]^ The remaining parameters for the cation were taken from the generalized OPLS force field for amines.[^49^, ^50^, ^51^] Partial charges were calculated from a gas phase restrained electrostatic potential (RESP)^[52]^ fit of the isolated ions at the HF/6‐31++G** level scaled down to an absolute value of 0.8.^[53]^ Charges are downscaled to account for the charge transfer and polarizability[^54^, ^55^, ^56^, ^57^, ^58^, ^59^] within ILs. However, the SPC/E[^60^, ^61^] model with unscaled charges was used for water in the IL‐water mixtures. The finally applied charges, as well as other parameters, can be found in the ESI. The initial boxes were generated using the PACKMOL^[62]^ program with the compositions as listed in Table 1. The LAMMPS^[63]^ program was used to carry out the classical MD simulations detailed herein at temperatures of 30 °C (303 K), 50 °C (323 K), 60 °C (333 K), and 80 °C (353 K) respectively. Cut‐offs for the Lennard‐Jones interactions were taken at 15 Å with tail corrections. For the evaluation of long‐range electrostatic interactions, we employed the standard Ewald sum technique^[64]^ with an accuracy of 10^−5^ Hartree beyond the cutoff distance. The timestep was set to 0.5 fs.

**Table 1:** The compositions and densities of the investigated systems. All densities are given in g mol^−1^. The experimental densities are given in parenthesis if available. Ball‐and‐stick representations of the ions are shown in Figure 1 and in the Supporting Information.

water content:	pure IL	0.1 %	1.0 %	2.0 %	3.8 %
[pyrH_4_] [NTf_2_] pairs	300	489	407	560	560
water molecules	–	11	93	256	512
density at					
30 °C	1.40 (1.40)	1.40 (1.40)	1.39 (1.39)	1.39 (1.39)	1.38 (1.38)
50 °C	1.38 (1.38)	1.38 (1.38)	1.37 (1.37)	1.37 (1.37)	1.36 (1.37)
60 °C	1.37 (1.37)	1.37 (1.37)	1.36 (1.36)	1.36 (−)	1.34 (−)
80 °C	1.34 (1.35)	1.34 (1.34)	1.34 (1.33)	1.33 (−)	1.32 (−)

All boxes were first equilibrated in an *NpT* ensemble at the specified target temperature and 1 bar for 2 ns. Thereby, the temperature and the pressure were maintained using a Nosé−Hoover thermostat and a Nosé−Hoover barostat, respectively.[^65^, ^66^, ^67^, ^68^] Afterwards, the boxes were compressed to result in the average size of the last 1 ns of these equilibrations. The obtained densities are listed in Table 1. The fact, that the densities perfectly match with the experimental ones validates the applied model.

The systems were reequilibrated for another 5 ns in a canonical ensemble. Afterwards, the production runs were performed for 10 ns in the microcanonical ensemble to ensure the unperturbated calculation of the Newtonian equations. Trajectories and thermodynamic information were saved every 250 steps for later analysis.

**Figure 1:**
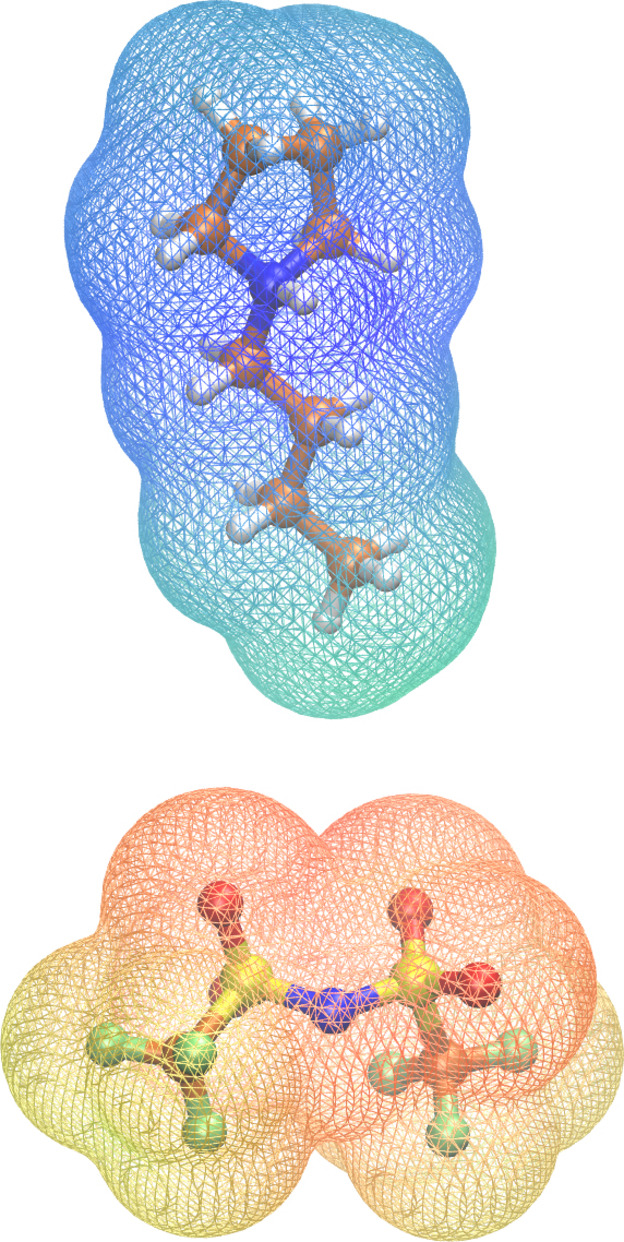
The ions of the investigated systems (top: cation, bottom: anion). The structures were optimized using the PBEh‐3c functional after which the electrostatic potential was mapped on the electronic density (isosurface value 0.0004). The color scheme goes from −0.2 (red) over 0 (green) to 0.2 (blue).

Static calculations were performed by the ORCA 4.0.1 program.[^69^, ^70^] The electrostatic potential shown in Figure 1 was calculated using the PBEh‐3c functional[^71^, ^72^, ^73^, ^74^, ^75^] after previous geometry optimization with the same method.

The VMD^[76]^ program was used for structure visualization. Trajectory analysis was performed by TRAVIS[^77^, ^78^] which allows for a wide variety of analyses to be performed from trajectory files. In this work, we evaluated radial distribution functions (RDFs), combined distribution functions (CDFs), diffusion coefficients and lifetimes using the reactive flux approach.[^79^, ^80^]

Self‐diffusion coefficients were calculated from the mean square displacements (MSDs) of the respective ions using the Einstein relation:

(1) $$D=\lim_{t\to \infty }\frac{1}{6t}\left\langle {\left|\vec{r}\left(t\right)-\vec{r}\left(0\right)\right|}^{2}\right\rangle$$

with the positions $\vec{r}\left(t\right)$ at time *t*. Only the data points in the last half of the corresponding mean squared displacement functions (shown in the Supporting Information) are considered for the linear regression.

Subsequently, the diffusion coefficients were used to calculate ionic mobilities using the Nernst‐Einstein relationship:

(2) $$\mu =\frac{D_{\mathrm{ions}}\cdot q}{k_{B}\cdot T}$$

with the self‐diffusion coefficient *D*_ions_, the charge *q*, the temperature *T*, and Boltzmann's constant *k*_B_.

We also performed a radical Voronoi tessellation,^[81]^ using TRAVIS, to understand the formation of phases, clusters and microheterogeneous fragments. Herein, the liquid is formally dissected into its building blocks in order to define different subsets;^[81]^ the definition of these subsets is system‐specific. All atoms are considered as Voronoi sites with the corresponding van der Waals radii being used to define borders, volumes and surfaces for each atom. The cells of each subset are formed from the atomic Voronoi cells and these initially defined subsets belong to the same domain if their cells share a common face. The average number of domains present in the liquid, *N*_Dom_ (domain count), can be obtained from such a formalism. Any value of *N*_Dom_ smaller than the total number of particular subsets that constitute it indicates a certain aggregation present in the system. If the domain count equals one, the subsets form a large, continuous microphase that stretches throughout the whole liquid.

## Results and Discussion

### Structure

**Figure 2:**
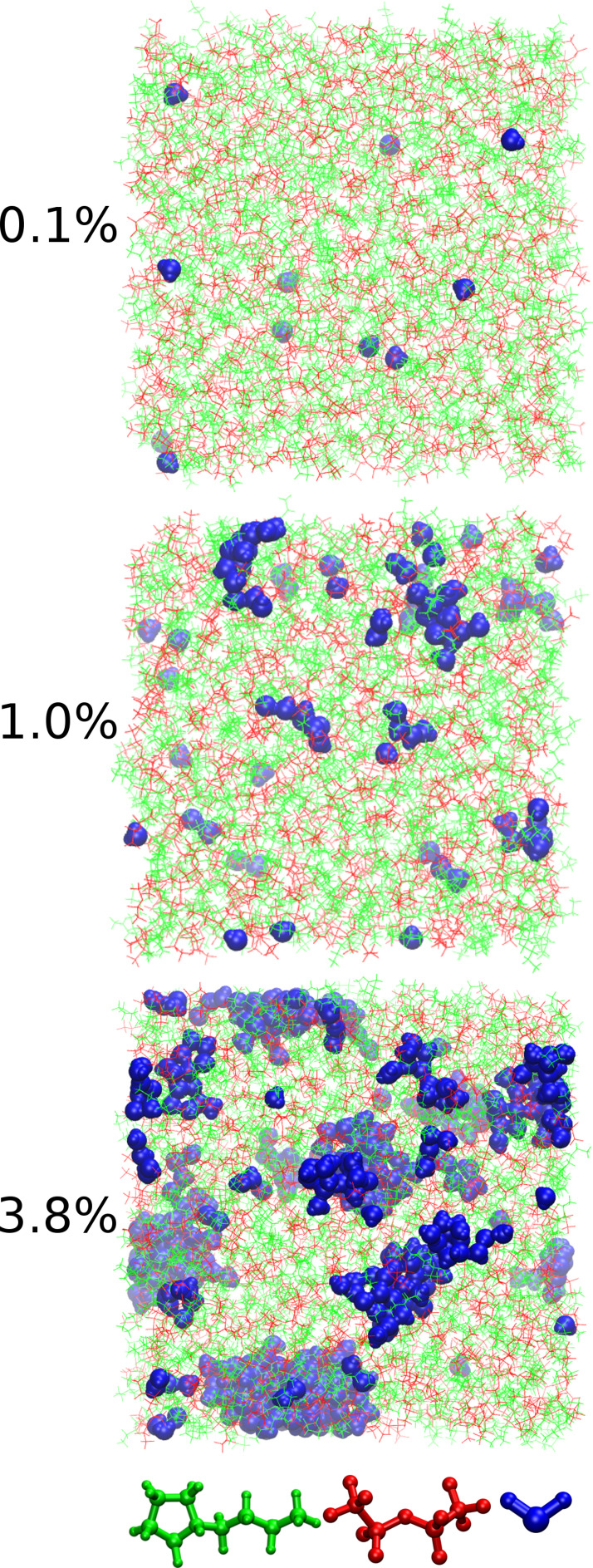
Snapshots from the simulations at 30 °C (303 K) for the 0.1 %, 1.0 % and 3.8 % water solutions. The cations, anions and water molecules are shown in green, red and blue, respectively.

The visualization of the trajectories, as illustrated by the representative snapshots in Figure 2, reveals an interesting distribution behavior of the added water. If the concentration of water is very low, the molecules are mostly found as single separated entities. With rising concentration, however, the water is increasingly clustering together to form big heterogeneous domains inside the liquid. It is justified to assume that a single water molecule clamped between ions shows a completely different behavior compared to a molecule in a small cluster or, even more extreme, to a molecule which is part of a bigger domain of quasi‐bulk water. In general, there are three possible states the water can exist in:

- as a shared solvent molecule being part of the solvation shell of two ions simultaneously
- as part of a solvation shell of a single ion
- as quasi‐bulk water.

In all these states, the physical properties of the water molecules should be significantly different. It should be noted here that the term “solvent” has to be taken with care. Especially for very low water content (see as an example the top panel of Figure 2), water is obviously not a solvent in the traditional sense of the term. More realistically spoken, water is a solute in the salt and should, therefore, be thought of something similar to crystal water in hydrate melts.

A robust way to quantify this cluster formation is the analysis of Voronoi domains.^[81]^ Therefore, we defined the following subsets based on the charge distribution of the electrostatic potentials shown in Figure 1:

- fluorous subset: trifluoromethyl groups of the anions
- aqueous subset: the water molecules
- non‐polar subset: the terminal propyl group of the cation
- polar subset: the rest of the cation and the anion

The resulting number of domains and the average respective domain sizes are shown in Table 2. Regardless of simulation temperature or concentration of water, the polar components are found to form a wide network detected as a single domain which is a well‐known observation for ionic liquids.[^82^, ^83^] Interestingly, the non‐polar as well as the fluorous parts are both characterized by a slight increase of the domain size – the number of molecules in one domain – if small amounts of water are added. If the amount of water increases above 2.0 %, however, the domain size increases rapidly which indicates the decomposition of the subset networks into smaller domains. Furthermore, for both subsets the domain numbers marginally decrease with increasing temperature. Meanwhile, for a water concentration of 0.1 % the water is well dispersed as mostly single molecules over the simulation cell in well separated domains. With 1.0 % water concentration, the average domain size increases to two molecules which indicates the appearance of small clusters. It has been shown that water clusters of small size form homodromic structures depending on their state.[^84^, ^85^] If the concentration of water is further increased, the clusters become bigger and increasingly dominating. A transition from small clusters (of two water molecules) to truly aggregated water molecules (with clusters of six and more molecules) is visible.

**Table 2:** Results of the domain analysis performed with the three terminal carbon atoms (and the attached hydrogen atoms) of the butyl chain as the non‐polar phase. The values give the number of respective separated domains. The average sizes of the respective domains are given in brackets since the simulations for different water concentrations were performed with varying numbers of ion pairs and water molecules.

	pure IL			
*T*	polar	non‐polar	fluorous	aqueous
30 °C	1.00 (300)	22.25 (13)	4.21 (71)	–
50 °C	1.00 (300)	21.17 (14)	4.20 (71)	–
60 °C	1.00 (300)	21.03 (14)	4.27 (70)	–
80 °C	1.00 (300)	19.69 (15)	4.12 (73)	–

	0.1 % water			
	polar	non‐polar	fluorous	aqueous
30 °C	1.00 (489)	35.06 (13)	6.46 (76)	10.52 (1)
50 °C	1.00 (489)	33.38 (14)	6.31 (77)	10.53 (1)
60 °C	1.00 (489)	32.43 (15)	6.30 (77)	10.33 (1)
80 °C	1.00 (489)	32.81 (15)	6.38 (77)	10.34 (1)

	1.0 % water			
	polar	non‐polar	fluorous	aqueous
30 °C	1.00 (407)	31.67 (13)	5.96 (68)	46.43 (2)
50 °C	1.00 (407)	31.31 (13)	5.83 (69)	47.43 (2)
60 °C	1.00 (407)	31.27 (13)	5.75 (70)	56.85 (2)
80 °C	1.00 (407)	30.02 (14)	5.83 (70)	55.54 (2)

	2.0 % water			
	polar	non‐polar	fluorous	aqueous
30 °C	1.00 (559)	44.60 (12)	8.61 (65)	41.61 (6)
50 °C	1.00 (559)	45.10 (12)	8.63 (65)	64.92 (4)
60 °C	1.00 (559)	45.75 (12)	8.57 (65)	76.27 (3)
80 °C	1.00 (559)	44.23 (12)	8.35 (67)	85.22 (3)

	3.8 % water			
	polar	non‐polar	fluorous	aqueous
30 °C	1.00 (559)	53.56 (10)	9.52 (58)	42.02 (12)
50 °C	1.01 (557)	49.47 (11)	9.42 (59)	54.81 (9)
60 °C	1.00 (557)	48.98 (11)	9.43 (59)	59.14 (9)
80 °C	1.01 (556)	48.17 (11)	9.75 (57)	85.59 (6)

**Figure 3:**
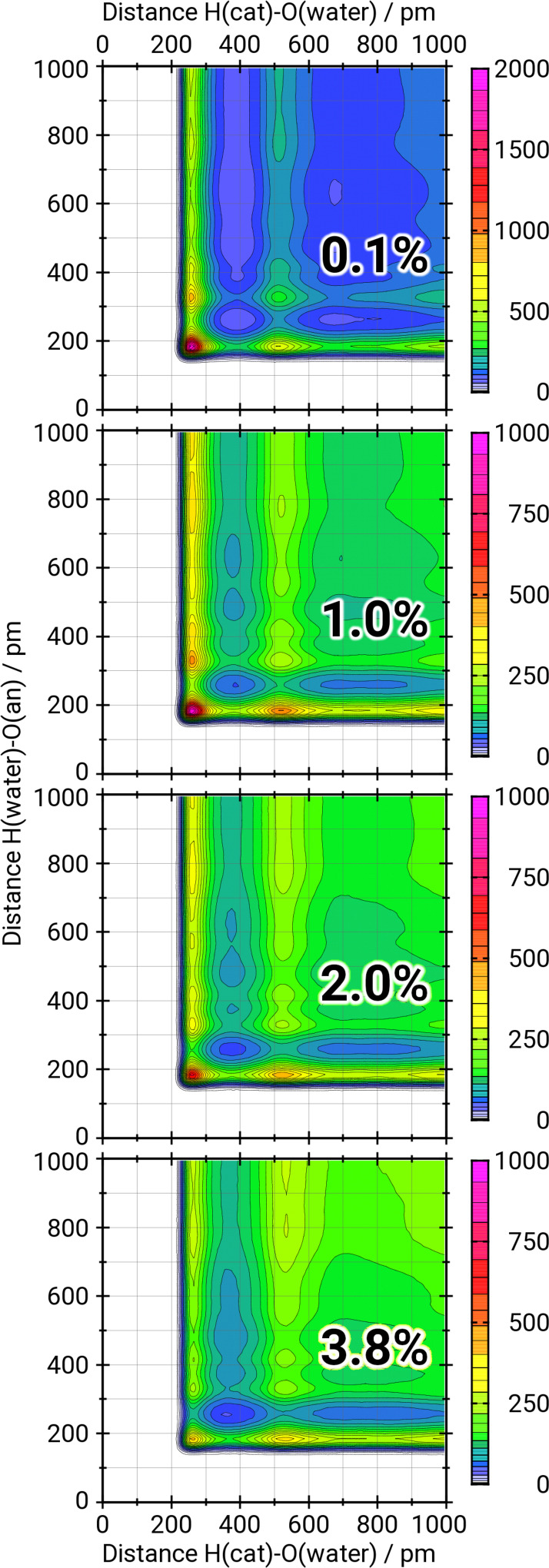
Combined distribution functions illustrating the occurrence of three body assemblies of a water molecule interaction with two ions simultaneously. The *x*‐axis gives the distance between the acidic hydrogen atom of the cation and the oxygen of the water molecule. The *y*‐axis gives the distance between the same water molecule's hydrogen atom and the oxygen atom of an anion. The heat plot represents the occurrence numbers of the certain assemblies. Note the different scaling in the top panel.

**Figure 4:**
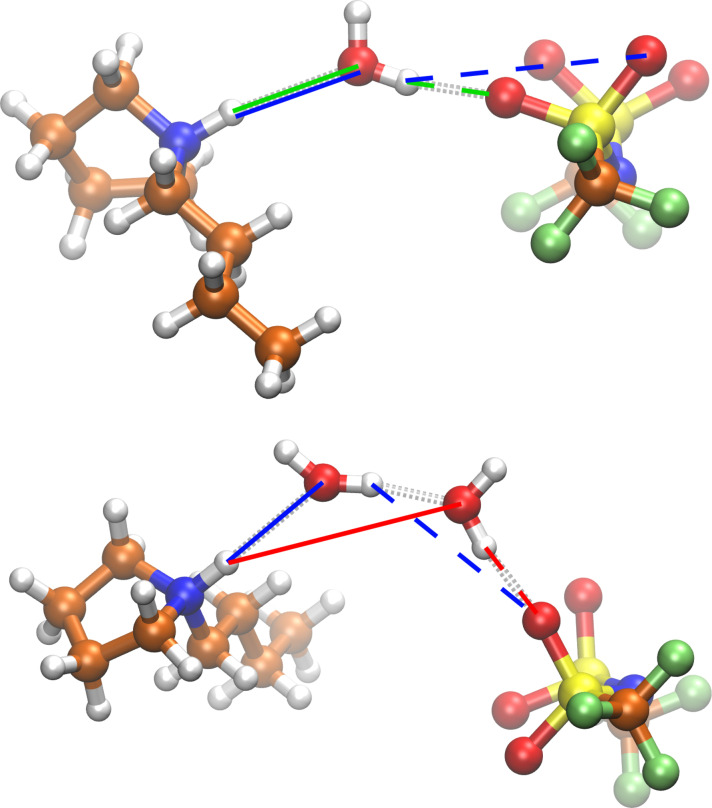
Most prominent assemblies found in the CDFs in Figure 3. The atoms are illustrated by the following color code: hydrogen (white), carbon (orange), nitrogen (blue), oxygen (red), fluorine (green), sulfur (yellow). Top: An anion and a cation separated by a single water molecule. Bottom: The two ions separated by a pair of water molecules. The relevant distances are marked with solid and dashed lines in blue, green, and red, respectively. See text for further explanation.

The combined distribution function (CDFs) in Figure 3 reveal the molecular details of the ion‐water interplay. Shown is the occurrence of assemblies of a water molecule at a certain distance from a cation and simultaneously at a given distance from an anion. For very low water concentrations, there is obviously only one prominent signal detected (named **A** in the following). The distances – about 185 pm for the water‐anion distance and about 255 pm for the cation‐water distance – are exactly those one would expect for the corresponding hydrogen bonds. Therefore, this peak can be assigned to the assembly illustrated in the top panel of Figure 4. The corresponding distances are marked by green lines. The two ions are separated by a water molecule in a way, that a hydrogen bond is formed between the cation and the water and another one between the same water and the anion. In other words, the water is in this case a shared solvent molecule.

With higher water content, two more signals become prominent:

- The first one (named **B**) is characterized by the same distance between water and anion as in **A** but by a distance of 515 pm between cation and water. This signal can be explained by the assembly illustrated in the bottom panel of Figure 4 and the distances marked by the red lines. In this case the two ions are separated by a bridge of two water molecules. If each of the water molecule is assigned to the solvation shell of one ion, this state represents two solvation shells interacting with each other.
- The second prominent signal (named **C**) is at the same distance between cation and water as in **A** but at a distance of 335 pm between water and anion. Due to the fact that the anion contains four hydrogen bond interaction sites, there are two possible explanations for this signal: First, the water exists as a shared solvent molecule but the signal is not representing the water‐anion distance of the interacting oxygen but, instead, of one of the other oxygen atoms (top panel of Figure 4, blue lines). Second, the water exists in two different interacting solvation spheres as in **B** (bottom panel of Figure 4, blue lines). Unfortunately, one has to act on the assumption that the signal contains contribution of both cases and is thereby degenerated.

Furthermore, in the CDF for the highest water content additional areas of high occurrence at higher distances in both dimensions are detected. These can be explained by the dominance of larger water clusters compared to the more distinct smaller clusters for the lower concentrations. Water molecules located in the inner region of larger clusters are not linked to any ion at all but only surrounded by other water molecules and thus comparable to molecules in a bulk water phase.

The qualitative interpretation of the CDFs in Figure 3 already reveals several interesting details. However, a quantitative interpretation is more challenging. Due to the different number of ions and water molecules as well as the different size of the simulation boxes and the consequential different normalization of the occurrence numbers, the direct comparison of different CDFs is invalid. Nevertheless, it is feasible to compare the ratio of two or more values taken from a single CDF with the corresponding ratios from another CDF.

**Figure 5:**
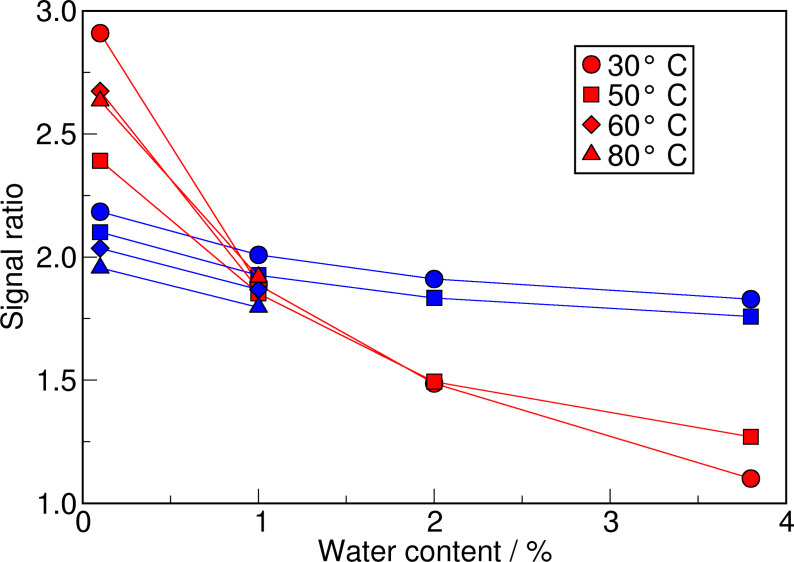
Evolution of the signal ratio with increasing water content. The ratio is given as the occurrence number of **A** divided by the occurrence number of **B** (red symbols) and **C** (blue symbols). The lines are a guide for the eye.

Due to the fact that the water can interact with the four oxygen atoms of the anion but only with the one acidic proton on the cation, even the comparison of the total numbers of the ratios **A : B** and **A : C** have to be handled with care. However, both ratios are clearly decreasing with increasing water concentration, as shown in Figure 5. Moreover, the slope of the **A : C** ratios is significantly less steep than for the **A : B** ratios. Another interesting observation is, that in the case of the **A : C** ratios the differences between different water concentrations are nearly the same for all temperatures, which becomes obvious if the eye‐guiding lines in Figure 5 are compared. For the **A : B** ratios this clear order is not observed.

### Ion pair and hydrogen bond dynamics

In Table 3 lifetimes of ions pairs and hydrogen bonds calculated using the reactive flux approach[^79^, ^80^] were summarized. As expected, all exchange rates increase with increasing temperature. Interestingly, the addition of water results in a similar effect.

**Table 3:** Ion‐pair and hydrogen bond lifetimes of the ion‐ion‐interactions calculated with the reactive flux approach. All values are given in ps.

Water content	*T*	IP	H(cation)−O(anion)
pure	30 °C	729.8	236.4
pure	50 °C	372.3	124.6
pure	60 °C	269.9	89.9
pure	80 °C	176.7	60.4
0.1 %	30 °C	697.3	224.4
0.1 %	50 °C	361.0	119.8
0.1 %	60 °C	236.0	89.1
0.1 %	80 °C	172.5	59.1
1.0 %	30 °C	479.6	188.7
1.0 %	50 °C	304.4	108.3
1.0 %	60 °C	223.4	83.5
1.0 %	80 °C	152.5	56.4
2.0 %	30 °C	546.4	178.8
2.0 %	50 °C	276.4	101.4
2.0 %	60 °C	220.4	81.5
2.0 %	80 °C	136.6	52.5
3.8 %	30 °C	468.8	172.4
3.8 %	50 °C	263.8	97.5
3.8 %	60 °C	207.1	78.3
3.8 %	80 °C	133.1	51.2

**Figure 6:**
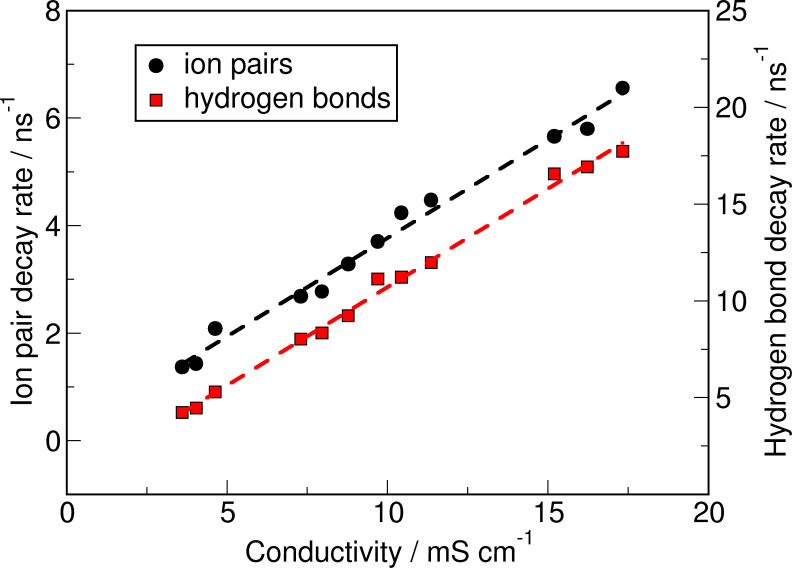
Relationship between the experimental conductivity^[47]^ and the inverse lifetimes (or decay rates) of hydrogen bonds and ion pairs.

Due to the fact, that the ion pair dynamics are directly related to the mobility of the ions the ion pair exchange rate can be correlated with the experimental conductivity,^[47]^ as visualized in Figure 6. A similar correlation can be observed for the exchange rate of the hydrogen bonds formed between cations and anions, demonstrating the importance of hydrogen bonding for the properties of ionic liquids. The revealed correlation between the calculated properties and the experimental data serves as a satisfying validation of the dynamical data.

The lifetimes of the water containing hydrogen bonds, listed in Table 4 reveal a similar behavior in terms of temperature dependence and water concentration as the ion pair dynamics. Interestingly, the lifetimes of the cation‐water hydrogen bonds are even slightly higher than those of the water‐water hydrogen bonds, except for the systems with 0.1 % water content. In contrast, the lifetimes of the water‐anion hydrogen bonds are significantly shorter. This observation renders the anion to be hydrophobic, while the cation's hydrophobic character is less pronounced.

**Table 4:** Hydrogen bond lifetimes of hydrogen bonds in between water molecules as well as water molecules and ions calculated with the reactive flux approach. All values are given in ps.

Water content	*T*	H(cation)−O(water)	H(water)−O(anion)	H(water)−O(water)
0.1 %	30 °C	338.5	37.4	590.7
0.1 %	50 °C	139.9	18.5	242.3
0.1 %	60 °C	98.9	13.4	141.2
0.1 %	80 °C	47.8	7.8	86.5
1.0 %	30 °C	174.0	28.0	87.2
1.0 %	50 °C	80.3	16.1	50.6
1.0 %	60 °C	73.2	11.0	39.9
1.0 %	80 °C	47.5	7.7	29.4
2.0 %	30 °C	132.4	23.9	41.1
2.0 %	50 °C	79.2	13.5	38.0
2.0 %	60 °C	57.3	9.1	35.9
2.0 %	80 °C	43.8	5.9	25.4
3.8 %	30 °C	103.3	19.2	28.2
3.8 %	50 °C	59.9	12.5	26.3
3.8 %	60 °C	42.8	9.0	24.4
3.8 %	80 °C	41.7	4.7	22.3

The observation that the lifetimes of the water‐water hydrogen bonds are longer than the corresponding cation‐water hydrogen bond lifetimes in the systems with 0.1 % water content can be understood if the underlying exchange process is taken into account: In the systems with a water concentration of more than 0.1 % clusters with more than two molecules are found. Therefore, it is possible that in the same step a water‐water hydrogen bond is broken a new water‐water hydrogen bond is formed. The high energetic stability of the new formed bond renders the barrier in this associative exchange mechanism to be smaller than a barrier towards a state of free partners in a dissociative mechanism.^[86]^ In contrast, the individual water molecules in the systems with 0.1 % water content rarely get in contact with each other. In other words, the hydrogen bonds exists in seldomly occurring water dimers, surrounded by the ionic liquid. As a consequence, the state of the former bonding partners after the breaking event has to be either the energetically unfavored free form, or a hydrogen bond with the ionic liquid. In the first case, the process needs to overcome the higher barrier of the dissociative mechanism. In the second case, it can be assumed, that the influence of these energetically less favorable hydrogen bonds on the barrier is weaker than the impact of the water‐water hydrogen bonds. Moreover, the concentration of the interaction sites in the IL is significantly lower than in water. Subsequently, the hydrogen bonds in the systems with 0.1 % water content are found to be longer living.

### Dipole reorientation

**Figure 7:**
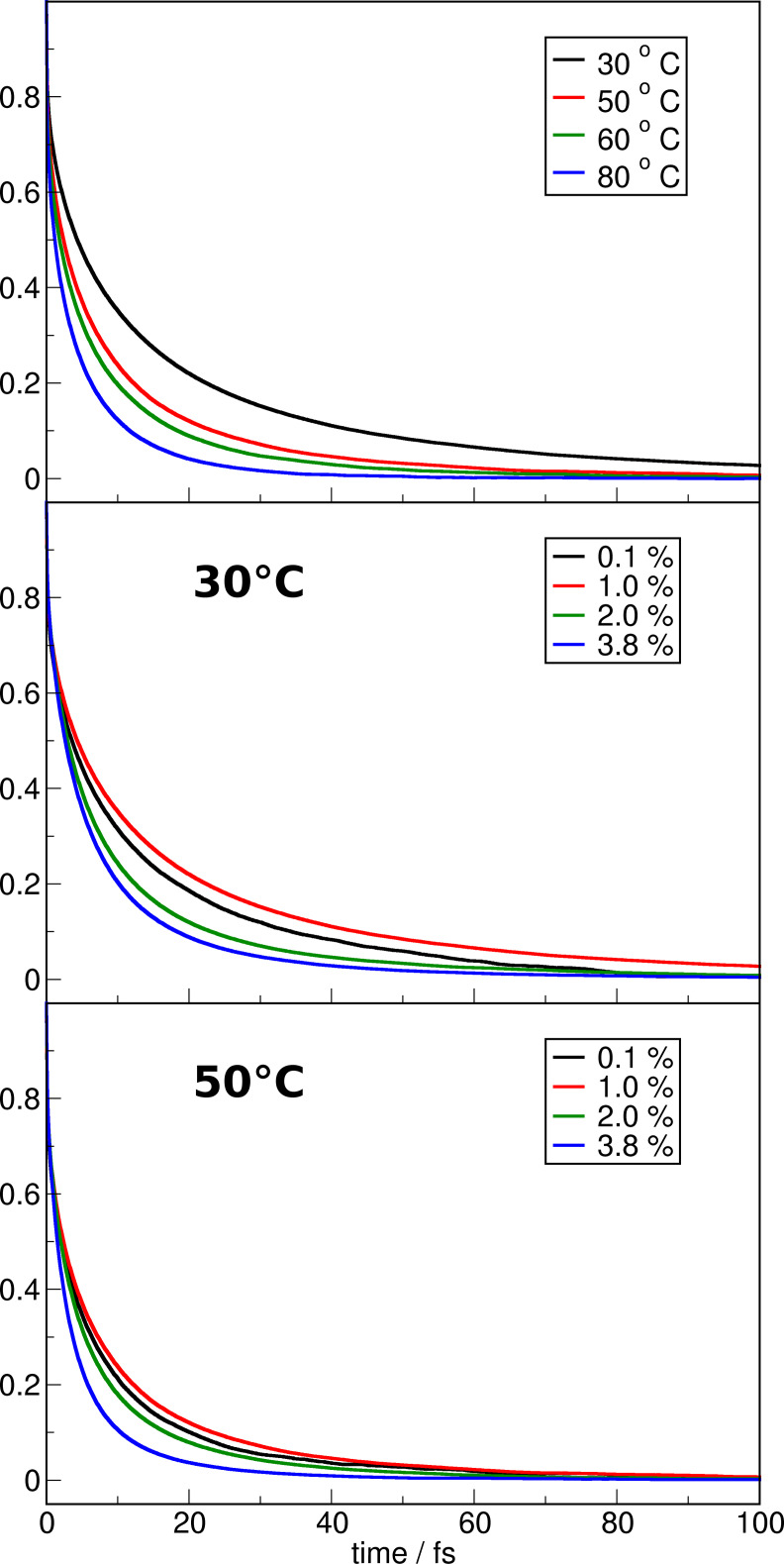
Water dipole reorientation dynamics in the water‐in‐IL solutions at different temperatures for the 1.0 % water solution (top panel) and for the different concentrations at 30 °C (middle panel) and 50 °C (bottom panel).

Figure 7 illustrates the dipole reorientation auto‐correlation functions of the water molecules. Similar to the observed trend for the ion pair and hydrogen bond dynamics the reorientation becomes faster if the temperature increases.

For the systems with a water content of 1.0 % and more a similar trend is observed if the concentration of water is increased. However, the systems with only 0.1 % water content do not follow this trend, but show at all temperatures a much faster reorientation than the corresponding 1.0 % systems. This is opposed to the trends in the hydrogen bond dynamics. Nonetheless, a similar line of argumentation can be used: The water is mostly found as single molecules in the systems with 0.1 % and is therefore only relatively weakly bound by hydrogen bonds with the surrounding ions. At higher concentrations the water molecules are forming small clusters and subsequently they are fixed in optimal cluster arrangements. If the water concentration is only at 1.0 % the found clusters consist of only 2–3 molecules, with strongly limited options to change the binding partner. Subsequently, the reorientation dynamics are slower. If the concentration increases the water's state changes more and more to that of bulk phase water, with a correspondingly rapid reorientation rate due to the fluctuating network of hydrogen bonds.

### Diffusion and ionic conductivity

Self‐diffusion coefficients are obtained from the Einstein relation using mean square displacements. Overall, the obtained diffusion coefficients are well comparable with diffusion coefficients (from both experiments and simulations) of several [NTf_2_]^−^‐based pure and doped ILs[^87^, ^88^, ^89^] and IL‐water systems.^[90]^

The trends shown in Table 5 reveal that at all investigated temperatures and all concentrations of water the cation exhibits faster diffusion than the anion. This was already observed in several [NTf_2_]^−^‐based ILs.[^88^, ^89^] Diffusion coefficients of all investigated species show a marked increase with temperature. Moreover, the diffusion coefficients of the ions increase as well with increasing water content with a few exceptions. These exceptions are presumably due to the fact that calculations of diffusion constants based on relatively short simulation times are known to lead to considerable uncertainties.^[91]^

**Table 5:** Self diffusion constants for the different simulations. All values are given in pm^2^ ps^−1^ and in the order cation/anion/water.

	30 °C	50 °C	60 °C	80 °C
pure	13.2/8.5/–	30.2/24.5/–	47.3/38.9/–	100.8/93.9/–
0.1 %	13.4/9.5/131.5	33.8/28.2/316.0	52.2/42.9/502.3	91.7/85.6/837.9
1.0 %	21.0/17.5/109.9	35.5/33.3/256.1	60.3/43.6/496.7	100.2/91.8/631.0
2.0 %	18.6/15.5/63.4	44.8/39.1/210.1	73.2/56.2/310.2	117.0/103.1/565.9
3.8 %	24.1/18.5/52.2	49.9/41.2/134.0	70.3/60.2/139.2	124.8/113.5/345.8

The observation that the ionic diffusion is sped up by the addition of water was already reported for imidazolium based PILs.^[92]^ However, the same work showed that the speed−up of the cation is larger than that of the anion. This is not the case for the pyrrolidinium based PIL investigated herein.

**Figure 8:**
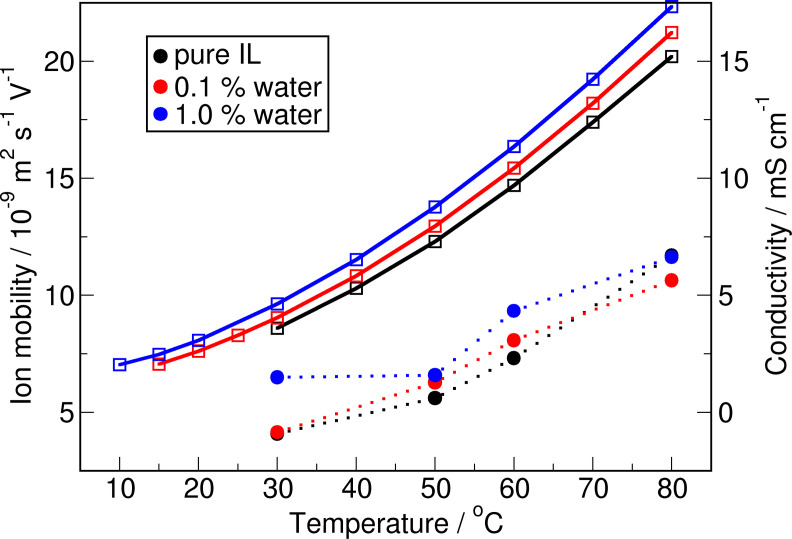
Ion mobility according to the Nernst‐Einstein‐relationship (circles and dotted lines) compared to the experimental conductivity^[47]^ (squares and solid lines). Both data sets show the same trends for the increasing temperature as well as for the increasing water content.

From the ionic mobility evaluated using the Nernst‐Einstein relationship with the obtained diffusivity coefficients shown in Table 6, we observe that the average ionic mobility increases marginally with addition of water but more significantly with increase in temperature. Actually, the observed trends resemble those of the experimental conductivity^[47]^ as illustrated in Figure 8, which provides a good validation of the presented dynamical data.

**Table 6:** The ionic mobility for the different systems in 10^−9^ m^2^ s^−1^ V^−1^ calculated according to the Nernst‐Einstein relationship. All values are given as cation /anion/average ionic mobility.

	30 °C	50 °C	60 °C	80 °C
pure	4.1/2.6/3.4	5.6/4.5/5.1	7.3/6.0/6.7	11.7/10.9/11.3
0.1 %	4.1/2.9/3.5	6.3/5.2/5.8	8.1/6.6/7.4	10.6/9.9/10.3
1.0 %	6.5/5.4/6.0	6.6/6.2/6.4	9.3/6.7/8.0	11.6/10.7/11.1
2.0 %	5.8/4.8/5.3	8.3/7.3/7.8	11.3/8.7/10.0	13.6/12.0/12.8
3.8 %	7.5/5.7/6.6	9.3/7.6/8.5	10.9/9.3/10.1	14.5/13.2/13.8

Additionally, the data in Table 5 reveals, that water generally exhibits a much higher diffusion coefficient than the ions. Interestingly, the diffusion coefficient of the water is not increasing with higher water content, but in contrast decreasing. Before, it was shown that the dynamics of the hydrogen bonds become faster with increasing concentration. The same trends were observed for the rotations of the water molecules. These accelerations co‐occur with an increase of the overall fluidity of the system in terms of the ion's diffusion coefficients and thus can be explained by a simple effect due to the dilution of the ionic liquid. This explanation is consistent with the recently published report, that the ion−ion interaction strength in PILs is not affected by the presence of water.^[93]^ Therefore, these observations alone do not allow an explicit statement about the state of the water.

The observed decrease of the translational mobility of the water molecules with increased concentration, however, shows an opposite trend compared to the system's fluidity. Hence, these findings clearly indicate that the water exists in different states within the ionic liquid depending on its concentration. While at low concentrations the water shows the typical behavior of a neutral molecule in an ionic liquid, the bulk water properties are more and more dominating if the concentration is increased. This is fully in line with the findings from the static analyses.

## Conclusions

The impact of water addition on the physical properties of the protic ionic liquid [pyrH4][NTf_2_] was reported previously.^[47]^ In this contribution, we complete these investigations by focusing the point of view on the physical state of the added water with the aid of molecular dynamics simulations. To achieve this goal, ionic liquids with different amounts of water additives were simulated at varying temperatures. In the analysis, generally, three different states of the water are conceptionally distinguished: First, water as a shared solvent molecule between two ions. Second, water as a member of a single solvent shell. And third, a state comparable to that in the bulk liquid.

Clear changes in the clustering behavior of the water molecules can already be identified by a simple visualization of the obtained trajectories and are further quantified by a Voronoi‐based domain analysis. Therein, it is found that for a water content of 0.1 % the water is well dispersed as single molecules. With a content of 1.0 % it is forming small clusters of two to three molecules. With higher contents, larger aggregates with average sizes of six and more molecules in averages are observed.

The molecular details of this clustering are illustrated with the aid of combined distribution functions. The simulations with 0.1 % water content is dominated by one prominent signal, which can be explained with water molecules simultaneously bound in two hydrogen bonds – one with an anion and one with a cation. In the above described concept, these water molecules are identified as shared solvents. At higher concentrations two more peaks arise. One is explained with water molecules which are members in two interacting single solvent shells. The other can be assumed as degenerated and is most probably the result of overlying contributions of shared solvent molecules and interacting single solvent shell molecules. The ratio of shared solvent to interacting solvent shell molecules is found to clearly decrease with increasing water content. Finally, for the highest concentrations, the occurrence at larger distances become more dominating which can be explained by the presence of a quasi‐bulk water microphase.

It is found that the decay rates of ion pairs and hydrogen bonds between cations and anions are correlated with the experimentally measured conductivities. Furthermore, the dynamics of both interactions become faster with increasing temperature as well as with increasing water content. Similar trends are observed for the reorientation of the water's dipoles. However, in this case an exception is found for the system with 0.1 % water content, which shows a faster reorientation than what one would expect. At this concentration the water molecules are present individually and therefore, relatively free to rotate. At higher concentrations the water molecules are hindered by the emanating water−water interactions. If the concentration increases the size of the formed clusters increase and subsequently the water's state is more approaching the state in the bulk phase and in consequence the dynamics become faster similar to the fluctuating hydrogen bond network.

Finally, the self diffusion coefficients calculated by the Einstein relation are analysed. It is shown, that all constants increase with increasing temperature. Furthermore, the coefficients of the ions increase with increasing water content. These trends are the same for the experimental conductivities.

Interestingly, the diffusion coefficients of the water molecules show the opposite trend corresponding the water content: The higher the water content is, the slower becomes the water's diffusion. The findings for the hydrogen bond dynamics and rotations of the water molecules can alternatively be explained as a side effect of the increase of the liquid's fluidity. However, the findings for the translations require a different explanation: For low concentrations the water behaves as a typical neutral solute in an ionic liquid. At higher concentrations this behavior is clearly due to a change in the water's state towards that in the bulk.

## Conflict of interest

The authors declare no conflict of interest.

## Supporting information

As a service to our authors and readers, this journal provides supporting information supplied by the authors. Such materials are peer reviewed and may be re‐organized for online delivery, but are not copy‐edited or typeset. Technical support issues arising from supporting information (other than missing files) should be addressed to the authors.

Supporting Information
