# Supplementary material for: Water in Protic Ionic Liquid Electrolytes: From Solvent Separated Ion Pairs to Water Clusters
Source: ChemSusChem. 2021 Jul 12;14(16):3315–24. doi: 10.1002/cssc.202100660 (PMC8456901; doi:10.1002/cssc.202100660)
Supplement: Supplementary file 1 — Supporting Information [file CSSC-14-3315-s001.pdf]

ChemSusChem

Supporting Information

Water in Protic Ionic Liquid Electrolytes: From Solvent Separated Ion Pairs to Water Clusters

Sascha Gehrke, Promit Ray, Timo Stettner, Andrea Balducci, and Barbara Kirchner*© 2021 The Authors. ChemSusChem published by Wiley-VCH GmbH. This is an open access article under the terms of the Creative Commons Attribution License, which permits use, distribution and reproduction in any medium, provided the original work is properly cited.

Contents

1 RESP Charges S2

2 Force-Field Parameters S4

3 Mean Square Displacements S6

1 RESP Charges

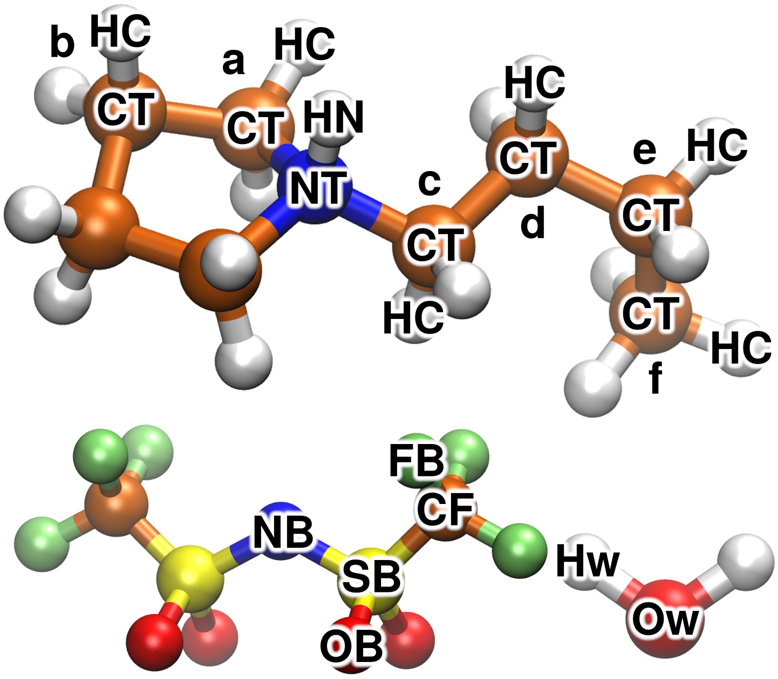

Figure S1: Atom types of the molecules applied in the simulations. Further, the CH_x groups of the cation are marked with letters from **a** to **f**.

Table 1: The scaled RESP charges applied in the article. For names of the atom types see S1.

Atom	Charge
$[NTf_2]^-$	
CF	0.18697643
FB	-0.10657503
NB	-0.51772659
OB	-0.43203516
SB	0.85568228
$[pyrH4]^+$	
CT a	-0.00941801
CT b	-0.06069608
CT c	-0.07591409
CT d	-0.03290304
CT e	0.00364400
CT f	-0.10874314
HC a	0.08150810
HC b	0.06301608
HC c	0.07849810
HC d	0.03382304
HC e	0.03298704
HC f	0.04195005
NT	-0.05826207
HN	0.21784327
water	
Ow	-0.8476
Hw	0.4238

2 Force-Field Parameters

```
    15 atom types
     9 bond types
    15 angle types
    13 dihedral types

Masses

   1     12.011000  # MolType 1 (C2F6NO4S2) CF
   2     18.998000  # MolType 1 (C2F6NO4S2) FB
   3     14.000000  # MolType 1 (C2F6NO4S2) NB
   4     15.999000  # MolType 1 (C2F6NO4S2) OB
   5     32.066000  # MolType 1 (C2F6NO4S2) SB
   6     12.011000  # MolType 2 (C8H18N   ) CT
   7     12.011000  # MolType 2 (C8H18N   ) CT
   8     12.011000  # MolType 2 (C8H18N   ) CT
   9     12.011000  # MolType 2 (C8H18N   ) CT
  10      1.008000  # MolType 2 (C8H18N   ) HN
  11      1.008000  # MolType 2 (C8H18N   ) HC
  12      1.008000  # MolType 2 (C8H18N   ) HC
  13     14.007000  # MolType 2 (C8H18N   ) NT
  14      1.008000  # MolType 3 (H2O      ) HW
  15     15.999000  # MolType 3 (H2O      ) OW

Pair Coeffs

   1      0.065999      3.500000  # MolType 1 (C2F6NO4S2) CF
   2      0.053000      2.950000  # MolType 1 (C2F6NO4S2) FB
   3      0.170000      3.250000  # MolType 1 (C2F6NO4S2) NB
   4      0.210000      2.960000  # MolType 1 (C2F6NO4S2) OB
   5      0.250000      3.550000  # MolType 1 (C2F6NO4S2) SB
   6      0.065999      3.500000  # MolType 2 (C8H18N   ) CT
   7      0.065999      3.500000  # MolType 2 (C8H18N   ) CT
   8      0.065999      3.500000  # MolType 2 (C8H18N   ) CT
   9      0.065999      3.500000  # MolType 2 (C8H18N   ) CT
  10      0.000000      0.000000  # MolType 2 (C8H18N   ) HN
  11      0.030000      2.500000  # MolType 2 (C8H18N   ) HC
  12      0.030000      2.500000  # MolType 2 (C8H18N   ) HC
  13      0.170000      3.250000  # MolType 2 (C8H18N   ) NT
  14      0.000000      0.000000  # MolType 3 (H2O      ) HW
  15      0.155354      3.166000  # MolType 3 (H2O      ) OW
```

```
Bond Coeffs

  1    233.030593      1.818000  # MolType 1 (C2F6NO4S2)  CF - SB
  2    441.921606      1.323000  # MolType 1 (C2F6NO4S2)  FB - CF
  3    374.880497      1.570000  # MolType 1 (C2F6NO4S2)  NB - SB
  4    637.069790      1.437000  # MolType 1 (C2F6NO4S2)  SB - OB
  5    382.002868      1.448000  # MolType 2 (C8H18N   )  NT - CT
  6    267.925430      1.529000  # MolType 2 (C8H18N   )  CT - CT
  7    339.985660      1.090000  # MolType 2 (C8H18N   )  HC - CT
  8    434.034417      1.010000  # MolType 2 (C8H18N   )  HN - NT
  9    450.000000      1.000000  # MolType 3 (H2O      )  OW - HW

Angle Coeffs

  1    82.934990    111.700000  # MolType 1 (C2F6NO4S2)  FB - CF - SB
  2    93.331740    107.100000  # MolType 1 (C2F6NO4S2)  FB - CF - FB
  3    80.186424    125.600000  # MolType 1 (C2F6NO4S2)  SB - NB - SB
  4    91.300191    103.500000  # MolType 1 (C2F6NO4S2)  NB - SB - CF
  5    94.287763    113.600000  # MolType 1 (C2F6NO4S2)  NB - SB - OB
  6   103.967495    102.600000  # MolType 1 (C2F6NO4S2)  CF - SB - OB
  7   115.798279    118.500000  # MolType 1 (C2F6NO4S2)  OB - SB - OB
  8    56.202199    109.500000  # MolType 2 (C8H18N   )  NT - CT - CT
  9    35.002390    109.500000  # MolType 2 (C8H18N   )  HC - CT - NT
 10    37.500000    110.700000  # MolType 2 (C8H18N   )  CT - CT - HC
 11    32.994742    107.800000  # MolType 2 (C8H18N   )  HC - CT - HC
 12    58.353250    112.700000  # MolType 2 (C8H18N   )  CT - CT - CT
 13    35.002390    109.500000  # MolType 2 (C8H18N   )  HN - NT - CT
 14    51.804493    107.200000  # MolType 2 (C8H18N   )  CT - NT - CT
 15    54.995220    109.470000  # MolType 3 (H2O      )  HW - OW - HW

Dihedral Coeffs

  1   7.832935  -2.490440  -0.763623   0.000000  # MolType 1 (C2F6NO4S2) SB - NB - SB - CF
  2   0.000000   0.000000   0.315966   0.000000  # MolType 1 (C2F6NO4S2) NB - SB - CF - FB
  3   0.000000   0.000000   0.346797   0.000000  # MolType 1 (C2F6NO4S2) OB - SB - CF - FB
  4   0.000000   0.000000  -0.003585   0.000000  # MolType 1 (C2F6NO4S2) OB - SB - NB - SB
  5   0.000000   0.000000   0.559990   0.000000  # MolType 2 (C8H18N   ) HC - CT - NT - CT
  6   0.415989  -0.128011   0.695005   0.000000  # MolType 2 (C8H18N   ) CT - NT - CT - CT
  7   1.300000  -0.050000   0.200000   0.000000  # MolType 2 (C8H18N   ) CT - CT - CT - CT
  8   0.000000   0.000000   0.300000   0.000000  # MolType 2 (C8H18N   ) CT - CT - CT - HC
  9  -0.190010  -0.416993   0.417997   0.000000  # MolType 2 (C8H18N   ) HN - NT - CT - CT
 10   2.391993  -0.673996   0.550000   0.000000  # MolType 2 (C8H18N   ) CT - CT - CT - NT
 11   0.000000   0.000000   0.400000   0.000000  # MolType 2 (C8H18N   ) HN - NT - CT - HC
 12   0.000000   0.000000   0.300000   0.000000  # MolType 2 (C8H18N   ) HC - CT - CT - HC
 13  -1.013002  -0.709011   0.472992   0.000000  # MolType 2 (C8H18N   ) HC - CT - CT - NT
```

3 Mean Square Displacements

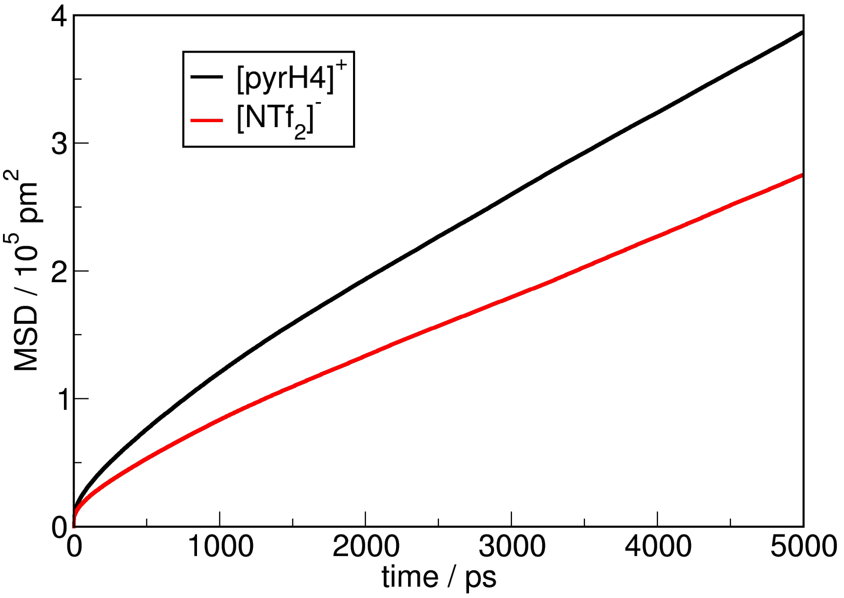

Figure S2: Mean square displacements calculated for the simulation of the pure ionic liquid at a temperature of 30° C.

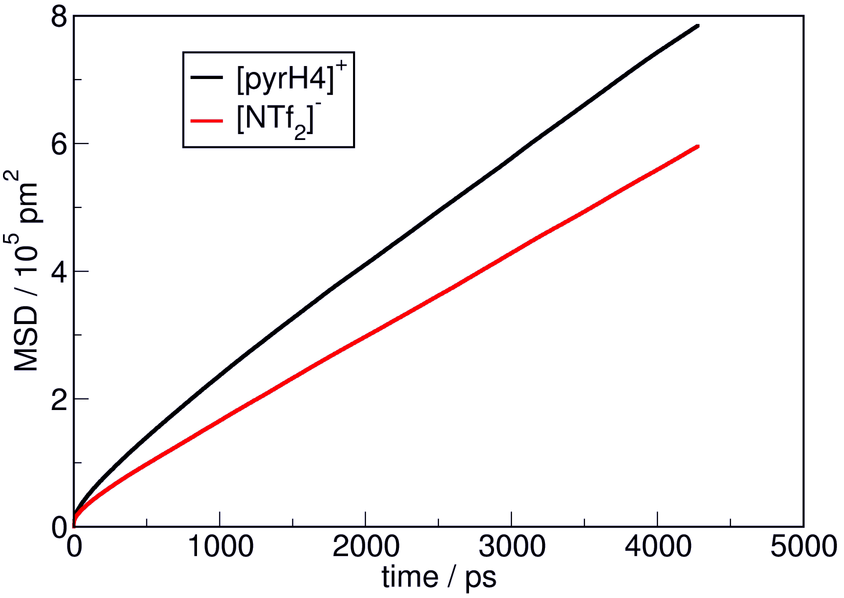

Figure S3: Mean square displacements calculated for the simulation of the pure ionic liquid at a temperature of 50° C.

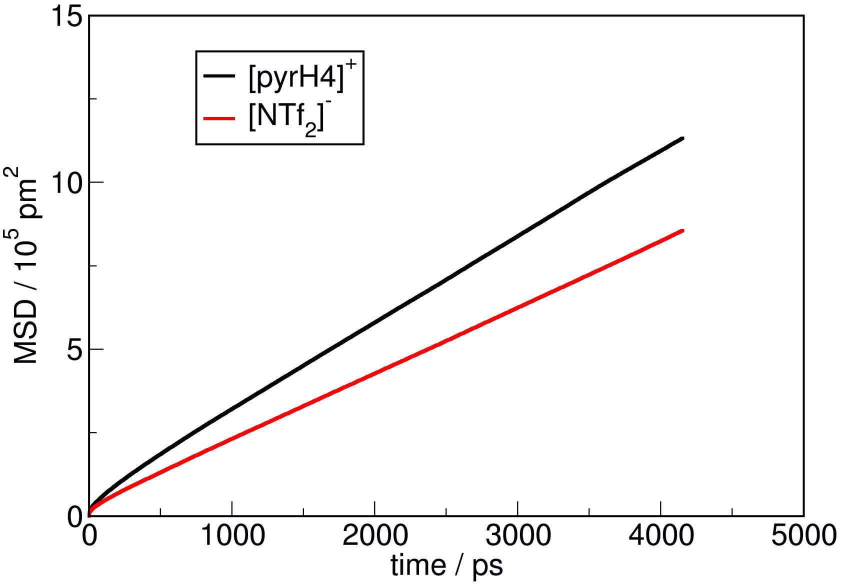

Figure S4: Mean square displacements calculated for the simulation of the pure ionic liquid at a temperature of 60° C.

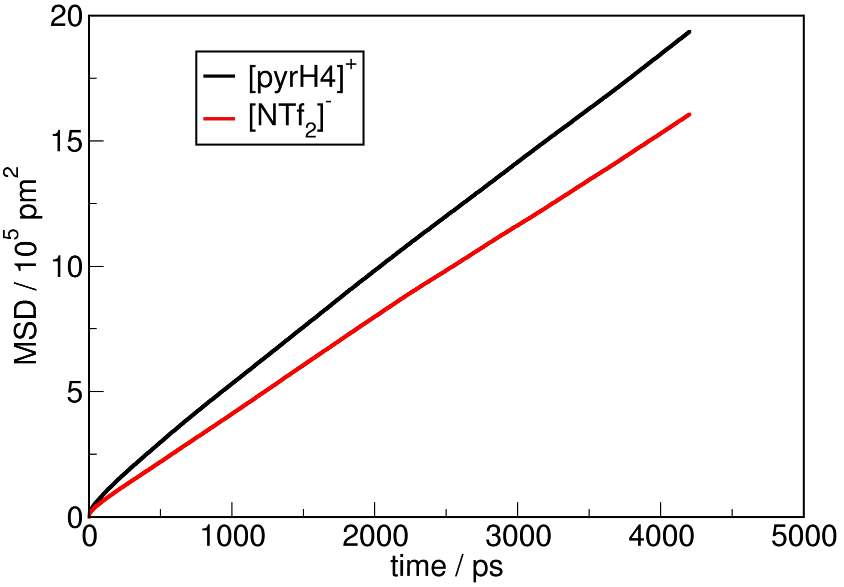

Figure S5: Mean square displacements calculated for the simulation of the pure ionic liquid at a temperature of 80° C.

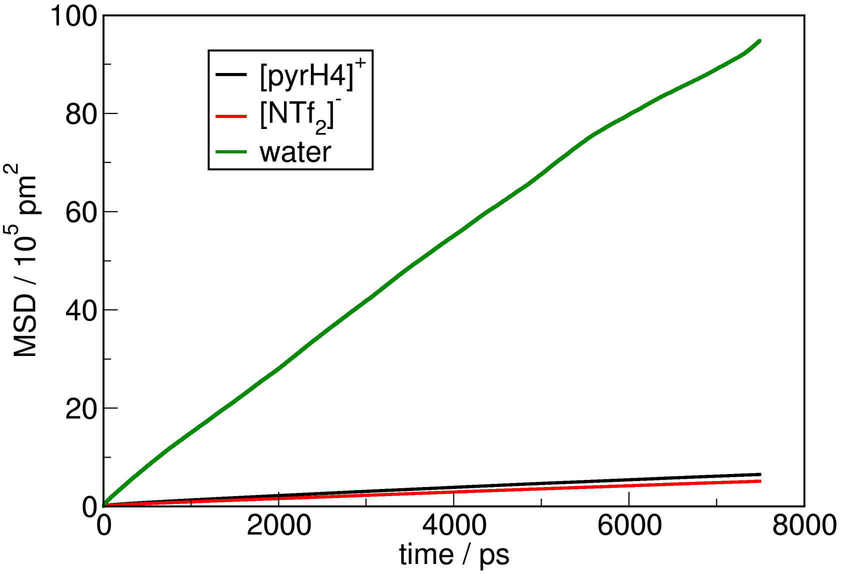

Figure S6: Mean square displacements calculated for the simulation of the ionic liquid with 0.1 % water at a temperature of 30° C.

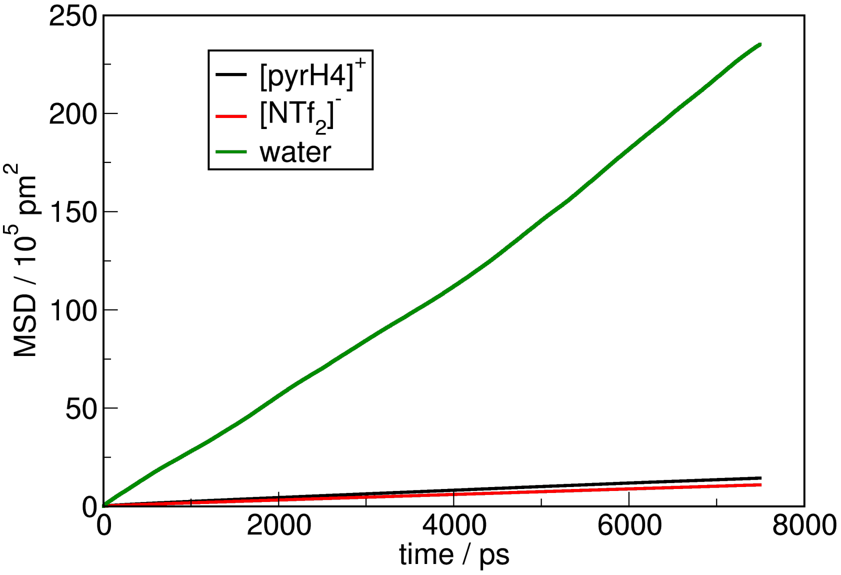

Figure S7: Mean square displacements calculated for the simulation of the ionic liquid with 0.1 % water at a temperature of 50° C.

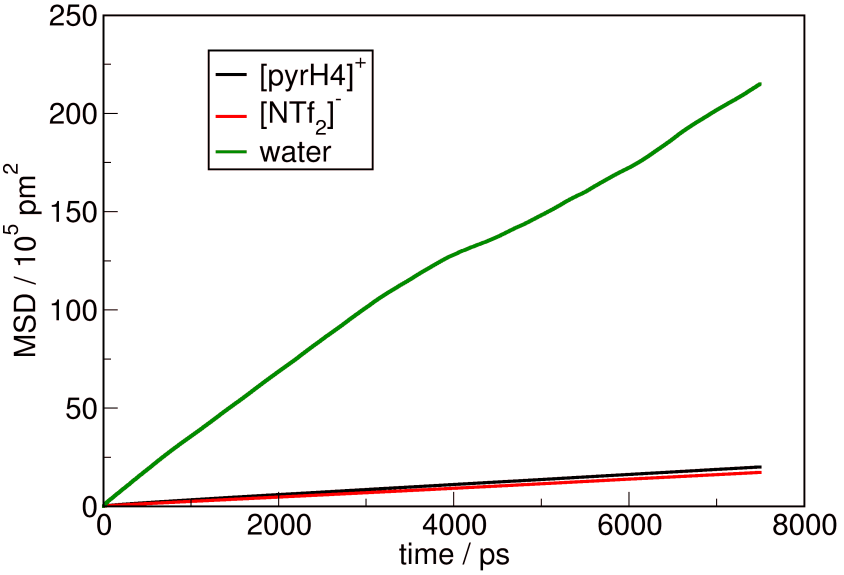

Figure S8: Mean square displacements calculated for the simulation of the ionic liquid with 0.1 % water at a temperature of 60° C.

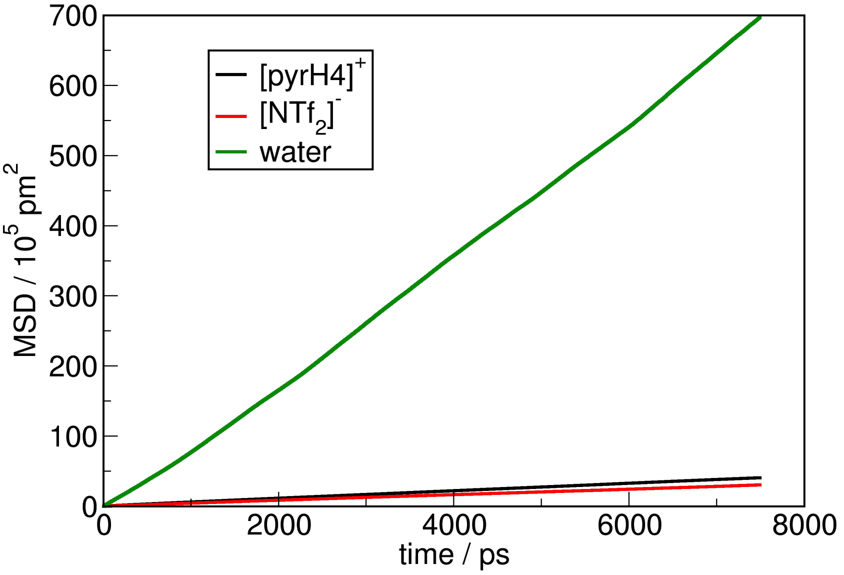

Figure S9: Mean square displacements calculated for the simulation of the ionic liquid with 0.1 % water at a temperature of 80° C.

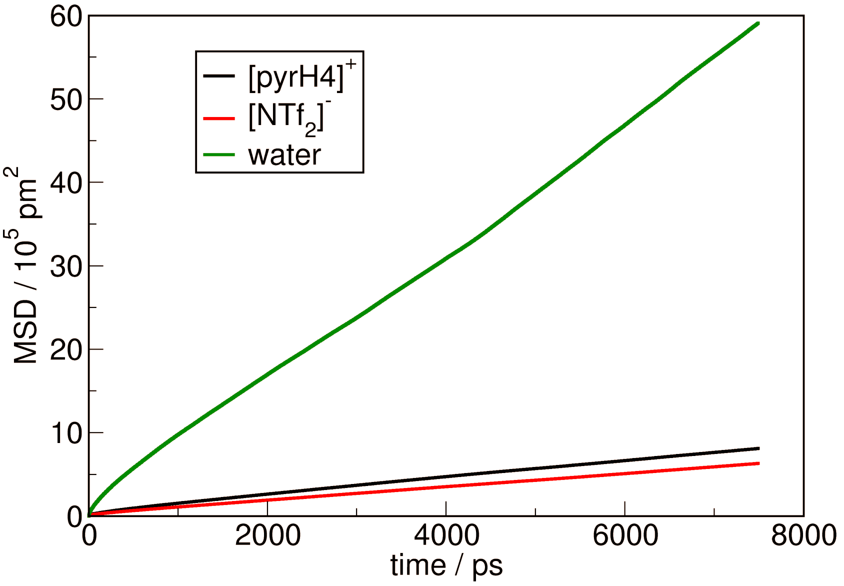

Figure S10: Mean square displacements calculated for the simulation of the ionic liquid with 1.0 % water at a temperature of 30° C.

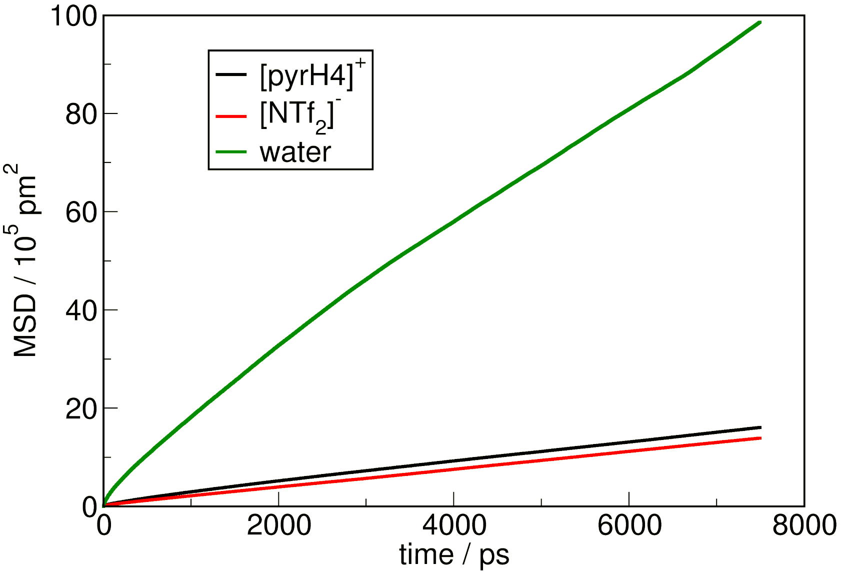

Figure S11: Mean square displacements calculated for the simulation of the ionic liquid with 1.0 % water at a temperature of 50° C.

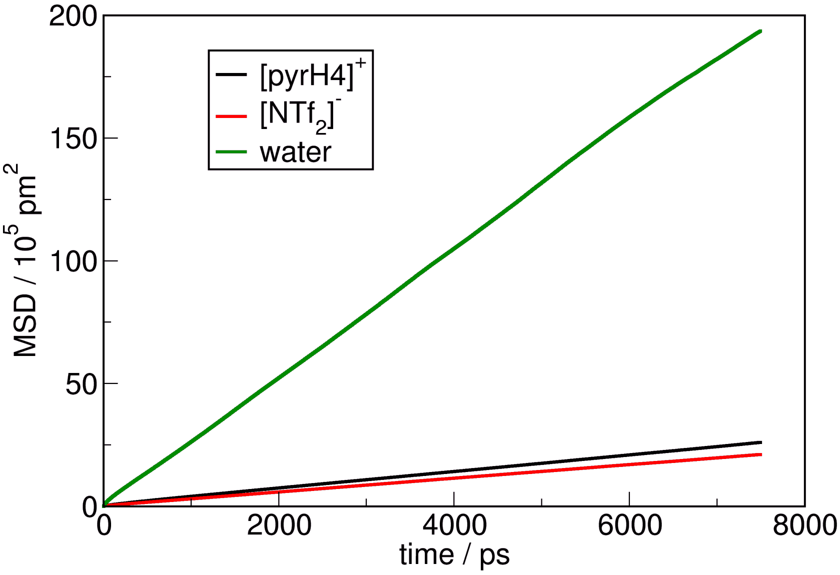

Figure S12: Mean square displacements calculated for the simulation of the ionic liquid with 1.0 % water at a temperature of 60° C.

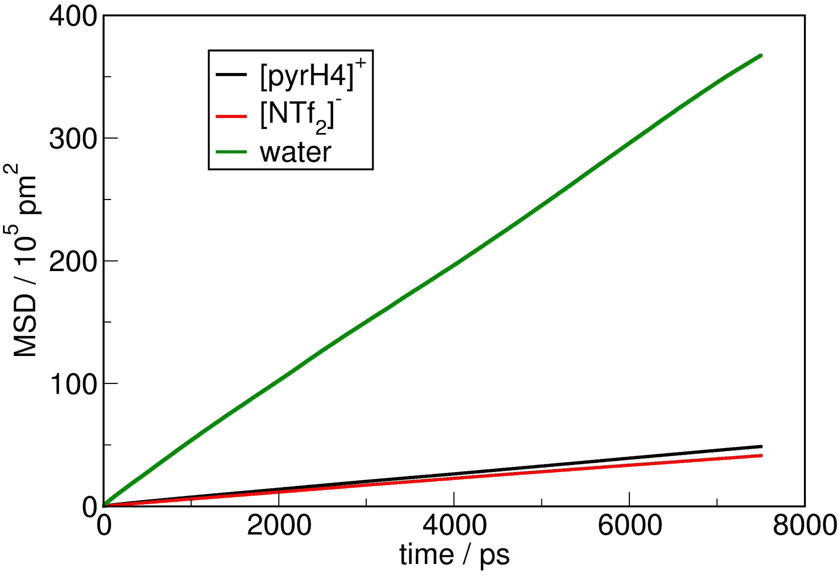

Figure S13: Mean square displacements calculated for the simulation of the ionic liquid with 1.0 % water at a temperature of 80° C.

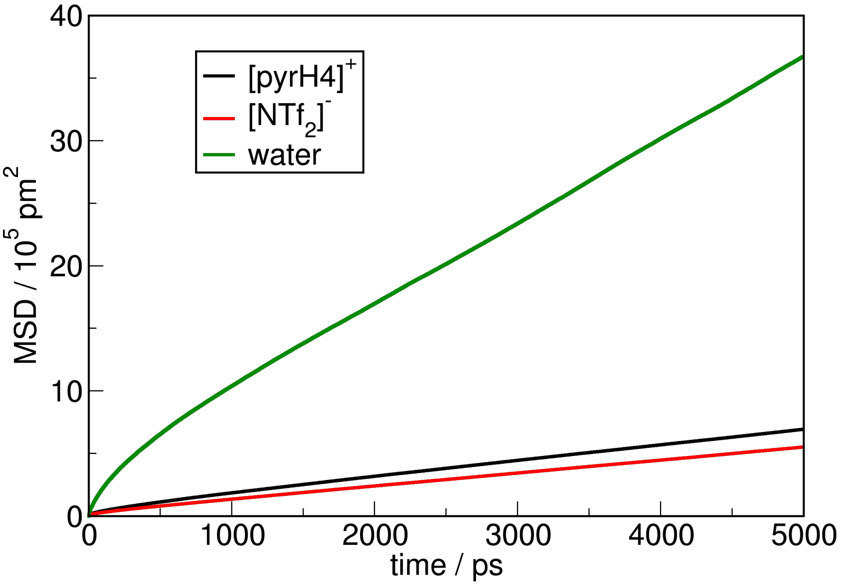

Figure S14: Mean square displacements calculated for the simulation of the ionic liquid with 2.0 % water at a temperature of 30° C.

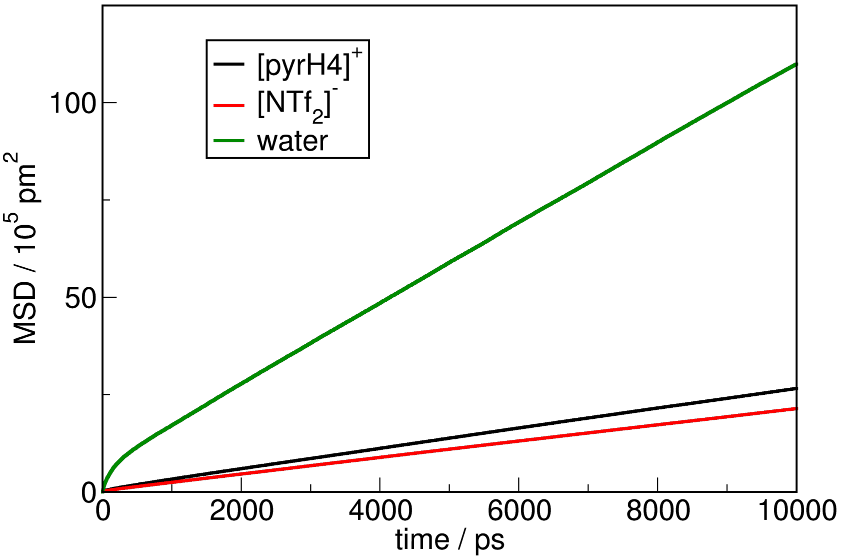

Figure S15: Mean square displacements calculated for the simulation of the ionic liquid with 2.0 % water at a temperature of 50° C.

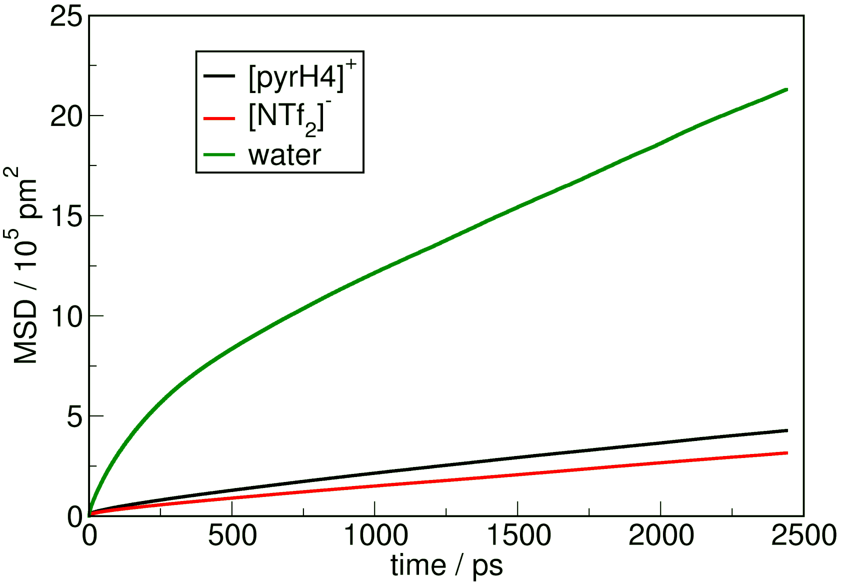

Figure S16: Mean square displacements calculated for the simulation of the ionic liquid with 3.8 % water at a temperature of 30° C.

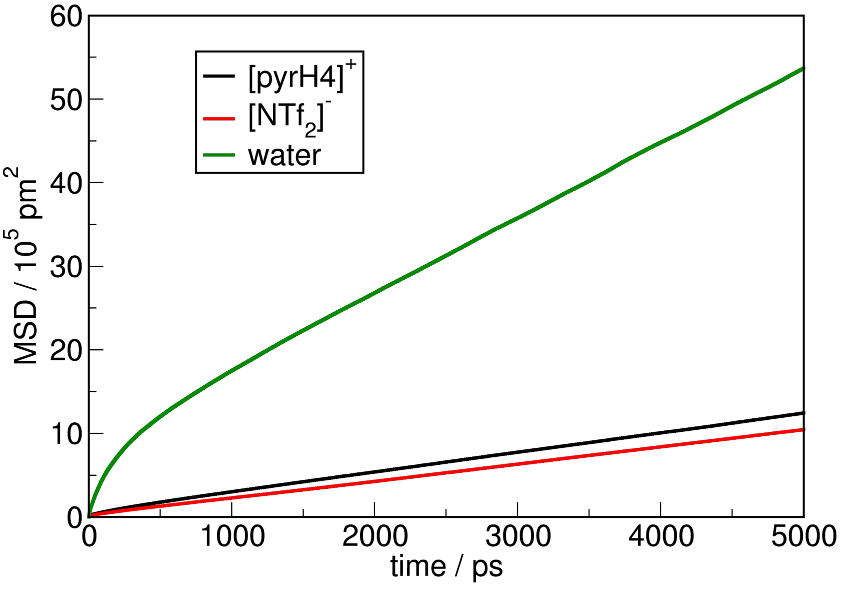

Figure S17: Mean square displacements calculated for the simulation of the ionic liquid with 3.8 % water at a temperature of 50° C.
